# Conformation and crystal structures of 1-amino­cyclo­hexa­neacetic acid (β^3,3^Ac_6_c) in N-protected derivatives^1^

**DOI:** 10.1107/S1600536814020777

**Published:** 2014-10-04

**Authors:** Naiem Ahmad Wani, Vivek K. Gupta, Rajni Kant, Subrayashastry Aravinda, Rajkishor Rai

**Affiliations:** a Medicinal Chemistry Division, Indian Institute of Integrative Medicine, Canal Road, Jammu Tawi 180 001, India; b X-ray Crystallography Laboratory, Post-Graduate Department of Physics & Electronics, University of Jammu, Jammu Tawi 180 006, India

**Keywords:** crystal structure, disubstituted-β-amino acids, π–π inter­action, hydrogen bonds, conformation

## Abstract

N-Protected derivatives of 1-amino­cyclo­hexa­neacetic acid (β^3,3^-Ac_6_c), namely Valeroyl-β^3,3^-Ac_6_c-OH [2-(1-pentanamidocyclohexyl)acetic acid, C_13_H_23_NO_3_], (I), Fmoc-β^3,3^-Ac_6_c-OH [2-(1-{[(9*H*-fluoren-9-yloxy)carbonyl]amino}cyclohexyl)acetic acid, C_23_H_25_NO_4_], (II), and Pyr-β^3,3^-Ac_6_c-OH {2-[1-(pyrazine-2-amido)cyclohexyl]acetic acid, C_13_H_17_N_3_O_3_}, (III), were synthesized and their conformational properties were determined by X-ray diffraction analysis. The backbone torsion angles (ϕ, θ) for β^3,3^-Ac_6_c-OH are restricted to *gauche* conformations in all the derivatives, with a chair conformation of the cyclo­hexane ring. In the crystal structure of (I), the packing of mol­ecules shows both carb­oxy­lic acid *R*_2_^2^(8) O—H⋯O and centrosymmetric *R* ^2^_2_(14) N—H⋯O hydrogen-bonding inter­actions, giving rise to chains along the *c*-axis direction. In (II), centrosymmetric carb­oxy­lic acid *R*_2_^2^(8) O—H⋯O dimers are extended through N—H⋯O hydrogen bonds and together with inter-ring π–π inter­actions between Fmoc groups [ring centroid distance = 3.786 (2) Å], generate a layered structure lying parallel to (010). In the case of compound (III), carb­oxy­lic acid O—H⋯N_pyrazine_ hydrogen bonds give rise to zigzag ribbon structures extending along the *c*-axis direction.

## Abstract 2

The *gauche* conformation of backbone torsion angles (ϕ, θ) for β^3^,-Ac_6_c-OH is observed in the N-protected derivatives of 1-amino­cyclo­hexa­neacetic acid.

## Chemical context   

**Figure 1:**
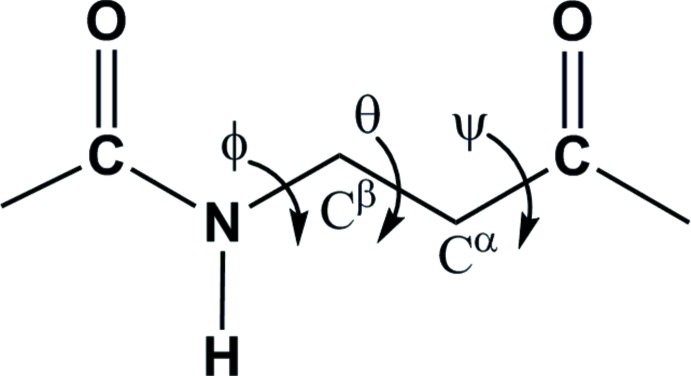
Definition of backbone torsion angles for β-amino acids.

β-Amino acids are homologues of α-amino acids, which are constituents of several bioactive natural and synthetic products. β-Amino acids have been used as building blocks in peptidomimetic drug design (Cheng *et al.* 2001 ▶). The introduction of β-amino acids into pharmacologically active peptide sequences has shown improved biological activity and metabolic stability (Yamazaki *et al.*, 1991 ▶; Huang *et al.*, 1993 ▶). The backbone conformation of a β-amino acid is defined by the torsional angles ϕ, θ and ψ (Banerjee & Balaram, 1997 ▶), as shown in Fig. 1 ▶. The monosubstitution at the α- and β-carbon atoms plays an important role in the folding of oligomers of β-amino acids (Seebach *et al.*, 2009 ▶). 
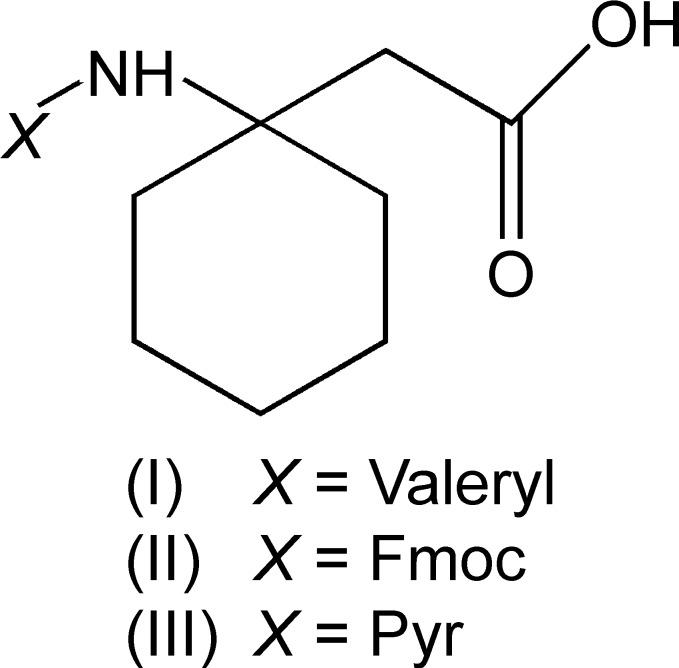


In order to investigate the effect of protecting groups and disubstitution on the conformation of β-amino acids, N-protected derivatives of 1-amino­cyclo­hexa­neacetic acid (β^3,3^Ac_6_c), *i.e.* Valeroyl-β^3,3^-Ac_6_c-OH (I), Fmoc-β^3,3^-Ac_6_c-OH (II) and Pyr-β^3,3^-Ac_6_c-OH (III) were synthesized. The crystal structures of the three compounds were determined and are reported herein, together with their comparative conformational features.
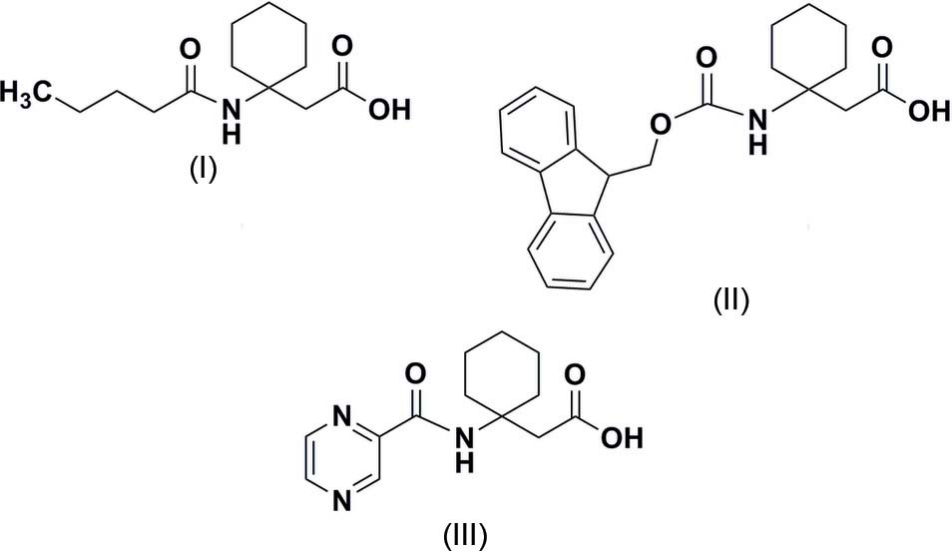


## Structural commentary   

**Figure 2:**
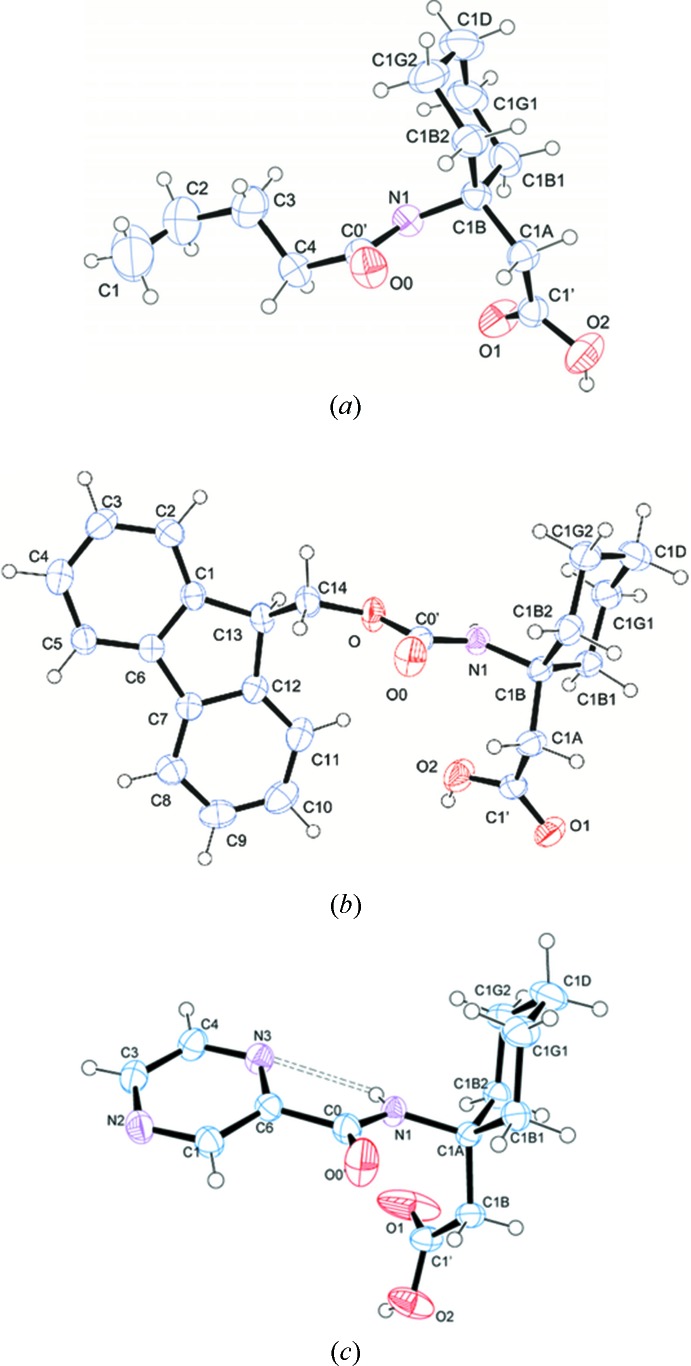
*ORTEP* view of the mol­ecular conformation with the atom-labelling scheme. for Valeroyl-β^3,3^-Ac_6_c-OH (I), (*b*) Fmoc-β^3,3^-Ac_6_c-OH (II) and (*c*) Pyr-β^3,3^-Ac_6_c-OH (III). The displacement ellipsoids are drawn at the 40% probability level. H atoms are shown as small spheres of arbitrary radii.

**Figure 3:**
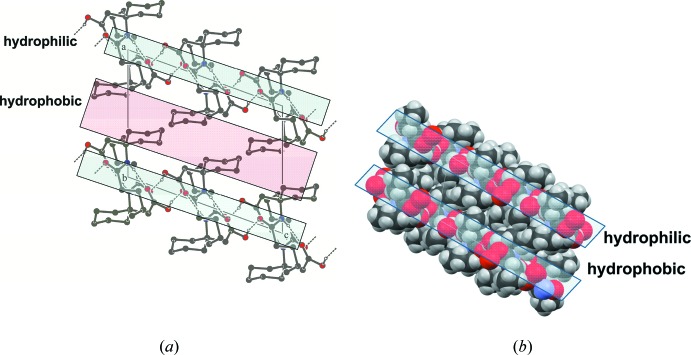
(*a*) Packing of Valeroyl-β^3,3^-Ac_6_c-OH (I) down the *b*-axis showing the alternative hydro­philic and hydro­phobic layers (*b*) space-filling model.

The mol­ecular conformations of Valeroyl-β^3,3^-Ac_6_c-OH (I), Fmoc-β^3,3^-Ac_6_c-OH (II) and Pyr-β^3,3^-Ac_6_c-OH (III) are shown in Fig. 2 ▶. The backbone torsion angles (ϕ, θ) (C0′—N1—C1*B* —C1*A* and N1—C1*B*—C1*A*—C1′) adopt a *gauche* conformation in all three compounds [ϕ = 61.9 (3)°, θ = 57.2 (3)° for (I); ϕ = 56.7 (3)°, θ = 66.1 (3)° for (II) and ϕ = 65.5 (2)°, θ = 55.0 (2)° for (III). The torsional angle ψ restricts the extended (*trans*) conformation for (I) [166.9 (2)°] and (III) [157.9 (2)°]. In the case of (II), it is restricted to a *gauche* conformation [*i.e.* ψ = −63.6 (3)°]. In a 3,3-disubstituted β-amino acid residue, β^3,3^-Ac_6_c-OH, the cyclo­hexane ring imposes a restriction on the torsion angles ϕ and θ. The protecting groups at the *N*-terminus of (I) adopts a *trans* geometry [ω_0_ (C4—C0′—N1—C1*B*) = 177.4 (2) for (I), ω_0_ (O—C0′—N1—C1*B*) = −175.64 (19) for (II) and ω_0_ (C6—C0—N1— C1*A*) = −170.04 (17)° for (III)]. In the case of the N-protected *tert*-butyl­oxycarbonyl (Boc) group, the protecting group adopts a *cis* geometry with ω_0_ = 14.50° (Vasudev *et al.*, 2008 ▶). The cyclo­hexane ring adopts a chair conformation with axial amino and equatorial CH_2_CO groups in all the derivatives. In Pyr-β^3,3^-Ac_6_c-OH (III), an intra­molecular N—H⋯N inter­action is observed between NH of the β^3,3^-Ac_6_c-OH residue and N3 of the pyrazine ring as shown in Fig. 3 ▶ *c*. There are no intra­molecular hydrogen bonding inter­actions observed in the crystal structures of derivatives (I) and (II).

## Supra­molecular features   

**Figure 4:**
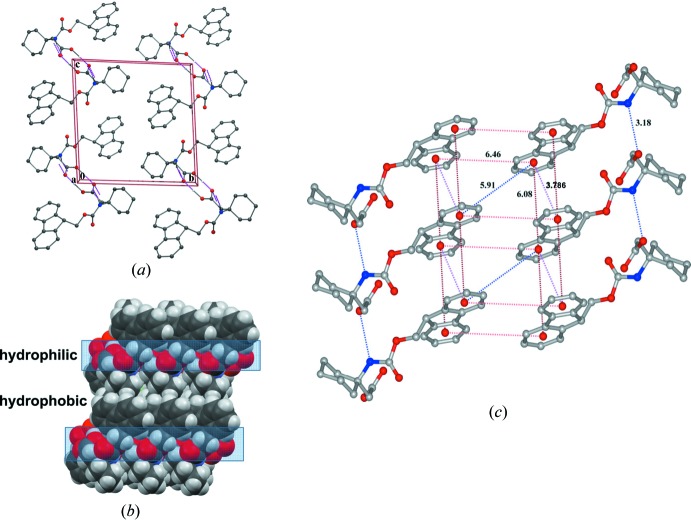
(*a*) Packing of Fmoc-β^3,3^-Ac_6_c-OH (II) down the *a*-axis. (*b*) Space-filling model showing the alternative hydro­philic and hydro­phobic layers (packing down the *c*-axis). (*c*) The environment of the Fmoc group showing the aromatic inter­action. The centroid–centroid distances are shown.

In the crystals of compounds (I) and (II), inter­molecular hydrogen-bonding inter­actions generate primary centrosymmetric dimeric but different substructures (Figs. 4 ▶ and 5 ▶). In (I), N1—H⋯O1^ii^ bond pairs (Table 1 ▶) give a cyclic 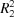
(14) motif which is extended into a ribbon structure along the *c*-axis direction through a second but non-centrosymmetric cyclic carb­oxy­lic acid 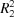
(8) O2—H⋯O^i^ hydrogen-bond motif (Fig. 4 ▶ *a*). In (II), the inter­molecular dimeric association is through the centrosymmetric 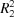
(8) carb­oxy­lic acid hydrogen-bonding motif. Structure extension is through N1—H⋯O1′ (carbox­yl) hydrogen bonds (Table 2 ▶), generating a two-dimensional layered structure lying parallel to (010) (Fig. 4 ▶ *c*). Also present in the structure are π–π inter­actions between the Fmoc groups with an inter­centroid distance of 3.786 (2) Å. Fig. 4 ▶ *c* shows the aromatic rings of Fmoc groups stacked in a face-to-face and edge-to-face manner, together with inter-plane distances that are within the range for stabilizing π–π inter­actions (Burley & Petsko, 1985 ▶; Sengupta *et al.*, 2005 ▶) and have been reported to induce self-assembly in peptides (Wang & Chau, 2011 ▶). In the case of (I) and (II), the mol­ecular packing in the crystals leads to the formation of alternating hydro­phobic and hydro­philic layers. In the crystals of (III), in which no dimer substructure formation is present, the mol­ecules are linked by an inter­molecular carb­oxy­lic acid O2—H⋯N2^i^ hydrogen bond (Table 3 ▶) with a pyrazine N-atom acceptor, leading to the formation of a zigzag ribbon structure extending along the *c*-axis direction.

## Synthesis and crystallization   

Preparation of Valeroyl-β^3,3^Ac_6_c-OH (I): β^3,3^Ac_6_c-OH (5 mmol, 785 mg) was dissolved in 5 ml of a 2*M* NaOH solution and a solution of 5 mmol of valeric anhydride (931 mg) dissolved in 1,4-dioxane was added, after which the mixture was stirred for 4 h at room temperature. On completion of the reaction, the 1,4-dioxane was evaporated and the product was extracted with diethyl ether (3 × 5 ml). The aqueous layer was acidified with 2*M* HCl and extracted with ethyl acetate (3 × 10ml) and the combined organic layer was washed with brine solution. The organic layer was passed over anhydrous Na_2_SO_4_ and evaporated to give Valeroyl-β^3^Ac_6_c-OH (yield: 1.1 g, 85.2%). Single crystals were grown by slow evaporation from a solution in methanol/water.

Preparation of Fmoc-β^3,3^Ac_6_c-OH (II): β^3,3^Ac_6_c-OH (10 mmol, 1.57 g) was dissolved in 1*M* Na_2_CO_3_ solution and Fmoc-OSu (10 mmol, 3.37 g) dissolved in CH_3_CN was added. The reaction mixture was stirred at room temperature for 6 h. After completion of the reaction, the CH_3_CN was evaporated and the residue was extracted with diethyl ether (3 × 10 ml). The aqueous layer was acidified with 2*M* HCl and extracted with ethyl acetate (3 × 15 ml). The combined organic layer was washed with brine solution. The ethyl acetate layer was passed over anhydrous Na_2_SO_4_ and evaporated. The residue was purified by crystallization in ethyl acetate/*n*-hexane, affording Fmoc-β^3,3^Ac_6_c-OH (yield: 3.0 g, 79%). Single crystals were obtained by slow evaporation from an ethyl acetate/*n*-hexane solution.

Preparation of Pyr-β^3,3^Ac_6_c-OH (III): Pyrazine carb­oxy­lic acid (3 mmol, 372 mg) was dissolved in dry CH_2_Cl_2_ and then 200 µl of *N*-methyl­morpholine was added, followed by β^3,3^Ac_6_c-OMe. HCl (3 mmol, 622.5 mg) and EDCI. HCl (3 mmol,576 mg) at 273 K. The reaction mixture was stirred at room temperature for 12 h. After completion of the reaction, water was added and the reaction mixture was extracted with CH_2_Cl_2_ (3 × 5ml). The combined organic layer was washed with 2*M* HCl (2 × 5ml), Na_2_CO_3_ (2 × 5ml) and brine solution (2 × 5ml). The organic layer was passed over anhydrous Na_2_SO_4_ and evaporated to give Pyr-β^3,3^Ac_6_c-OMe (Yield: 600 mg, 72.2%). Pyr-β^3,3^Ac_6_c-OMe (2 mmol, 554 mg) was dissolved in 2 ml of methanol and 1 ml of 2*M* NaOH, and the reaction mixture was stirred at room temperature for 4 h. Methanol was evaporated and the residue was extracted with diethyl ether (2 × 5ml). The aqueous layer was acidified with 2*M* HCl and extracted with ethyl acetate (3 × 5ml). The combined organic layer was washed with brine solution (1 × 5ml). The ethyl acetate layer was passed over anhydrous Na_2_SO_4_ and evaporated to give Pyr-β^3,3^Ac_6_c-OH (yield: 370 mg, 70.3%). Single crystals were grown from an ethanol/water solution.

## Refinement details   

Crystal data, data collection and structure refinement details are summarized in Table 4 ▶. For derivative (I), H atoms for N1 and O2 were located in a difference Fourier map and both their coordinates and *U*_iso_ values were refined. The remaining H atoms were positioned geometrically and were treated as riding on their parent C atoms, with C—H distances of 0.96–0.98 Å and with *U*_iso_(H) = 1.2*U*_eq_(C) or 1.5*U*_eq_(methyl C). For derivatives (II) and (III), all hydrogen atoms were located from a difference Fourier map and both their coordinates and *U*_iso_ values were refined. In (II), the carboxyl O—H distance was constrained to 0.84 Å. Although not of consequence with the achiral mol­ecule of (III), which crystallized in the non-centrosymmetric space group *Pca*2_1_, the structure was inverted in the final cycles of refinement as the Flack parameter was 0.8 (14). The inverted structure gave a value of 0.2 (14) for 1585 Friedel pairs.

## Supplementary Material

Crystal structure: contains datablock(s) I, II, III, New_Global_Publ_Block. DOI: 10.1107/S1600536814020777/zs2313sup1.cif

Structure factors: contains datablock(s) I. DOI: 10.1107/S1600536814020777/zs2313Isup2.hkl

Structure factors: contains datablock(s) II. DOI: 10.1107/S1600536814020777/zs2313IIsup3.hkl

Structure factors: contains datablock(s) III. DOI: 10.1107/S1600536814020777/zs2313IIIsup4.hkl

Supporting information file. DOI: 10.1107/S1600536814020777/zs2313Isup5.cml

Supporting information file. DOI: 10.1107/S1600536814020777/zs2313IIsup6.cml

Supporting information file. DOI: 10.1107/S1600536814020777/zs2313IIIsup7.cml

CCDC references: 1024488, 1024489, 1024490

Additional supporting information:  crystallographic information; 3D view; checkCIF report

## Figures and Tables

**Figure 5:**
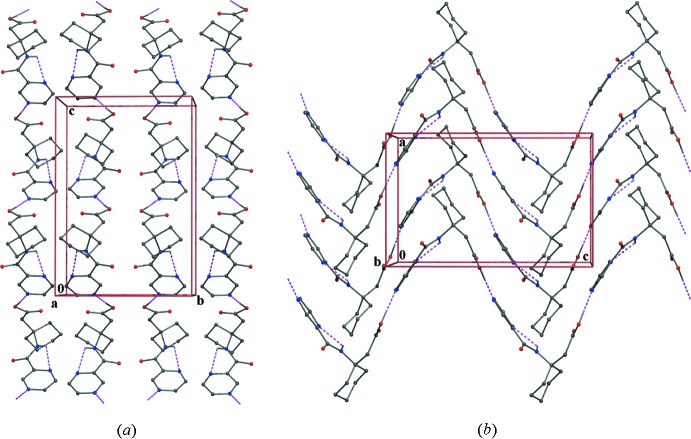
(*a*) Packing of Pyr-β^3,3^-Ac_6_c-OH (III) down the *a*-axis showing the ribbon structure. (*b*) Zigzag arrangement of the ribbons along the *c*-axis.

**Table 1:** Hydrogen-bond geometry (, ) for (I)

*D*H*A*	*D*H	H*A*	*D* *A*	*D*H*A*
O2H2*O*O0^i^	0.87(4)	1.74(4)	2.599(3)	166(4)
N1H1*N*O1^ii^	0.82(3)	2.16(3)	2.981(3)	172(2)

Symmetry codes: (i) 
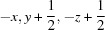
; (ii) 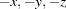.

**Table 2:** Hydrogen-bond geometry (, ) for (II)

*D*H*A*	*D*H	H*A*	*D* *A*	*D*H*A*
N1H1*N*O1^i^	0.86(2)	2.35(2)	3.182(3)	161(2)
O2H2*O*O1^ii^	0.84(3)	1.83(3)	2.673(3)	177(1)

Symmetry codes: (i) 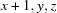; (ii) 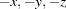.

**Table 3:** Hydrogen-bond geometry (, ) for (III)

*D*H*A*	*D*H	H*A*	*D* *A*	*D*H*A*
O2H21N2^i^	0.93(4)	1.86(4)	2.791(3)	177(4)
N1H1*N*N3	0.79(2)	2.34(2)	2.729(2)	111.3(19)

Symmetry code: (i) 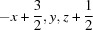.

**Table 4:** Experimental details

	(I)	(II)	(III)
Crystal data			
Chemical formula	C_13_H_23_NO_3_	C_23_H_25_NO_4_	C_13_H_17_N_3_O_3_
*M*_r_	241.32	379.44	263.30
Crystal system, space group	Monoclinic, *P*2_1_/*c*	Triclinic, *P* 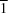	Orthorhombic, *P* *c* *a*2_1_
Temperature (K)	291	291	291
*a*, *b*, *c* ()	9.5894(5), 12.5007(7), 12.3709(8)	6.0834(4), 12.7642(9), 12.8399(9)	8.7135(1), 10.5321(1), 14.3907(2)
, , ()	90, 109.984(7), 90	94.018(6), 92.295(6), 100.489(6)	90, 90, 90
*V* (^3^)	1393.66(14)	976.53(12)	1320.66(3)
*Z*	4	2	4
Radiation type	Mo *K*	Mo *K*	Mo *K*
(mm^1^)	0.08	0.09	0.10
Crystal size (mm)	0.30 0.08 0.08	0.30 0.05 0.03	0.25 0.25 0.25

Data collection			
Diffractometer	Oxford Diffraction Xcalibur, Sapphire3 CCD	Oxford Diffraction Xcalibur, Sapphire3 CCD	Oxford Difraction Xcalibur, Sapphire3 CCD
Absorption correction	Multi-scan (*CrysAlis PRO*; Oxford Diffraction, 2010 ▶)	Multi-scan (*CrysAlis PRO*; Oxford Diffraction, 2010 ▶)	Multi-scan (*CrysAlis PRO*; Oxford Diffraction, 2010 ▶)
*T*_min_, *T*_max_	0.797, 1.000	0.947, 1.000	0.931, 1.000
No. of measured, independent and observed [*I* > 2(*I*)] reflections	14087, 2737, 1717	7781, 4166, 2037	68869, 2878, 2670
*R*_int_	0.047	0.047	0.034
(sin /)_max_ (^1^)	0.617	0.639	0.639

Refinement			
*R*[*F* ^2^ > 2(*F* ^2^)], *wR*(*F* ^2^), *S*	0.068, 0.213, 1.03	0.054, 0.086, 0.97	0.042, 0.106, 1.04
No. of reflections	2737	4166	2878
No. of parameters	162	353	240
No. of restraints	0	1	1
H-atom treatment	H atoms treated by a mixture of independent and constrained refinement	All H-atom parameters refined	All H-atom parameters refined
_max_, _min_ (e ^3^)	0.36, 0.30	0.15, 0.20	0.27, 0.26
Absolute structure			(Flack, 1983 ▶): 1585 Friedel pairs
Absolute structure parameter			0.2(14)

Computer programs: *CrysAlis PRO* (Oxford Diffraction, 2010 ▶), *SHELXS97* and *SHELXL97* (Sheldrick, 2008 ▶), *ORTEP-3 for Windows* (Farrugia, 2012 ▶) and *PLATON* (Spek, 2009 ▶).

## supplementary crystallographic information

### Crystal data

**Table:**

C_13_H_17_N_3_O_3_	*F*(000) = 560
*M**_r_* = 263.30	*D*_x_ = 1.324 Mg m^−^^3^
Orthorhombic, *P**c**a*2_1_	Mo *K*α radiation, λ = 0.71073 Å
Hall symbol: P 2c -2ac	Cell parameters from 28796 reflections
*a* = 8.7135 (1) Å	θ = 3.7–27.0°
*b* = 10.5321 (1) Å	µ = 0.10 mm^−^^1^
*c* = 14.3907 (2) Å	*T* = 291 K
*V* = 1320.66 (3) Å^3^	Cube, colorless
*Z* = 4	0.25 × 0.25 × 0.25 mm

### Data collection

**Table:**

Oxford Difraction Xcalibur, Sapphire3 CCD diffractometer	2878 independent reflections
Radiation source: fine-focus sealed tube	2670 reflections with *I* > 2σ(*I*)
Graphite monochromator	*R*_int_ = 0.034
Detector resolution: 16.1049 pixels mm^-1^	θ_max_ = 27.0°, θ_min_ = 3.9°
ω scans	*h* = −11→11
Absorption correction: multi-scan (*CrysAlis PRO*; Oxford Diffraction, 2010)	*k* = −13→13
*T*_min_ = 0.931, *T*_max_ = 1.000	*l* = −18→18
68869 measured reflections	

### Refinement

**Table:**

Refinement on *F*^2^	Secondary atom site location: difference Fourier map
Least-squares matrix: full	Hydrogen site location: difference Fourier map
*R*[*F*^2^ > 2σ(*F*^2^)] = 0.042	All H-atom parameters refined
*wR*(*F*^2^) = 0.106	*w* = 1/[σ^2^(*F*_o_^2^) + (0.048*P*)^2^ + 0.4913*P*] where *P* = (*F*_o_^2^ + 2*F*_c_^2^)/3
*S* = 1.04	(Δ/σ)_max_ = 0.032
2878 reflections	Δρ_max_ = 0.27 e Å^−^^3^
240 parameters	Δρ_min_ = −0.26 e Å^−^^3^
1 restraint	Absolute structure: (Flack, 1983): 1585 Friedel pairs
Primary atom site location: structure-invariant direct methods	Absolute structure parameter: 0.2 (14)

### Special details

**Table:**

Experimental. *CrysAlis PRO*, Oxford Diffraction Ltd., Version 1.171.34.40 (release 27–08-2010 CrysAlis171. NET) (compiled Aug 27 2010,11:50:40) Empirical absorption correction using spherical harmonics, implemented in SCALE3 ABSPACK scaling algorithm.
Geometry. All esds (except the esd in the dihedral angle between two l.s. planes) are estimated using the full covariance matrix. The cell esds are taken into account individually in the estimation of esds in distances, angles and torsion angles; correlations between esds in cell parameters are only used when they are defined by crystal symmetry. An approximate (isotropic) treatment of cell esds is used for estimating esds involving l.s. planes.
Refinement. Refinement of F^2^ against ALL reflections. The weighted R-factor wR and goodness of fit S are based on F^2^, conventional R-factors R are based on F, with F set to zero for negative F^2^. The threshold expression of F^2^ > 2sigma(F^2^) is used only for calculating R-factors(gt) etc. and is not relevant to the choice of reflections for refinement. R-factors based on F^2^ are statistically about twice as large as those based on F, and R- factors based on ALL data will be even larger.

### Fractional atomic coordinates and isotropic or equivalent isotropic displacement parameters (Å^2^)

**Table:**

	*x*	*y*	*z*	*U*_iso_*/*U*_eq_	
H1	0.902 (2)	0.412 (2)	1.0592 (15)	0.030 (5)*	
H1B2	1.400 (3)	0.435 (2)	1.2329 (16)	0.041 (6)*	
H1B5	1.272 (3)	0.384 (2)	1.4161 (18)	0.049 (7)*	
H1B6	1.183 (3)	0.442 (2)	1.3342 (17)	0.048 (6)*	
H1B1	1.499 (2)	0.380 (2)	1.3191 (16)	0.033 (5)*	
H1D2	1.651 (3)	0.100 (3)	1.200 (2)	0.061 (7)*	
H1N	1.161 (2)	0.172 (2)	1.2259 (16)	0.036 (6)*	
H1G1	1.481 (3)	0.256 (2)	1.1391 (19)	0.045 (6)*	
H2	0.709 (3)	0.107 (2)	0.971 (2)	0.051 (7)*	
H3	0.894 (3)	−0.021 (2)	1.0429 (17)	0.047 (6)*	
H1B4	1.269 (3)	0.107 (2)	1.3669 (17)	0.045 (6)*	
H1G4	1.401 (3)	0.050 (3)	1.232 (2)	0.061 (7)*	
H1G2	1.616 (3)	0.323 (3)	1.173 (2)	0.066 (8)*	
H1B3	1.415 (3)	0.192 (2)	1.3989 (19)	0.046 (6)*	
H1G3	1.502 (3)	0.005 (3)	1.3184 (19)	0.065 (8)*	
H1D1	1.667 (4)	0.176 (3)	1.298 (2)	0.071 (9)*	
H21	0.885 (5)	0.352 (4)	1.458 (3)	0.106 (13)*	
C1	0.9028 (2)	0.3259 (2)	1.05370 (16)	0.0401 (4)	
N2	0.7893 (2)	0.27249 (19)	1.00502 (14)	0.0442 (4)	
C3	0.7875 (3)	0.1459 (2)	1.00191 (15)	0.0433 (5)	
C4	0.8954 (3)	0.0736 (2)	1.04730 (16)	0.0450 (5)	
N3	1.0074 (2)	0.12584 (16)	1.09722 (13)	0.0398 (4)	
C6	1.0105 (2)	0.25193 (18)	1.10026 (14)	0.0343 (4)	
C0	1.1318 (2)	0.31669 (18)	1.15876 (15)	0.0382 (4)	
O0'	1.1604 (2)	0.42890 (15)	1.14626 (15)	0.0658 (6)	
N1	1.19610 (18)	0.24153 (15)	1.22264 (12)	0.0348 (4)	
C1A	1.2995 (2)	0.28157 (17)	1.29864 (13)	0.0311 (4)	
C1B	1.2100 (2)	0.36660 (19)	1.36671 (16)	0.0381 (4)	
C1'	1.0648 (2)	0.31013 (19)	1.40619 (15)	0.0423 (5)	
O1	1.0357 (3)	0.2011 (2)	1.4129 (3)	0.1197 (13)	
O2	0.9700 (2)	0.39599 (18)	1.43574 (16)	0.0677 (6)	
C1B1	1.4369 (2)	0.3562 (2)	1.26078 (16)	0.0394 (4)	
C1G1	1.5373 (3)	0.2777 (2)	1.19617 (18)	0.0504 (5)	
C1D	1.5948 (3)	0.1582 (3)	1.2448 (2)	0.0575 (6)	
C1B2	1.3586 (2)	0.15966 (19)	1.34564 (14)	0.0366 (4)	
C1G2	1.4634 (3)	0.0817 (2)	1.28311 (18)	0.0483 (5)	

### Atomic displacement parameters (Å^2^)

**Table:**

	*U*^11^	*U*^22^	*U*^33^	*U*^12^	*U*^13^	*U*^23^
C1	0.0386 (10)	0.0391 (10)	0.0428 (10)	0.0036 (9)	−0.0049 (9)	0.0025 (10)
N2	0.0374 (8)	0.0538 (11)	0.0414 (9)	0.0036 (8)	−0.0067 (7)	0.0039 (8)
C3	0.0402 (11)	0.0513 (13)	0.0386 (11)	−0.0063 (9)	−0.0045 (9)	−0.0018 (10)
C4	0.0510 (12)	0.0399 (11)	0.0440 (10)	−0.0049 (10)	−0.0066 (9)	−0.0016 (9)
N3	0.0445 (9)	0.0361 (8)	0.0389 (9)	−0.0002 (7)	−0.0048 (8)	0.0035 (7)
C6	0.0340 (9)	0.0368 (10)	0.0321 (9)	0.0012 (7)	0.0002 (7)	0.0023 (8)
C0	0.0388 (10)	0.0335 (9)	0.0422 (11)	0.0024 (8)	−0.0048 (8)	0.0032 (8)
O0'	0.0769 (12)	0.0360 (8)	0.0843 (13)	−0.0095 (8)	−0.0359 (11)	0.0160 (8)
N1	0.0365 (8)	0.0291 (8)	0.0388 (9)	−0.0040 (6)	−0.0074 (7)	0.0016 (6)
C1A	0.0299 (8)	0.0305 (9)	0.0329 (9)	−0.0015 (7)	0.0010 (7)	−0.0030 (7)
C1B	0.0352 (10)	0.0327 (10)	0.0464 (11)	−0.0002 (8)	0.0058 (9)	−0.0069 (8)
C1'	0.0418 (11)	0.0358 (10)	0.0494 (12)	−0.0009 (9)	0.0134 (9)	−0.0037 (9)
O1	0.0886 (16)	0.0440 (10)	0.227 (3)	−0.0088 (10)	0.099 (2)	−0.0111 (15)
O2	0.0533 (10)	0.0462 (9)	0.1035 (15)	0.0071 (8)	0.0373 (10)	0.0115 (9)
C1B1	0.0322 (9)	0.0395 (11)	0.0464 (11)	−0.0066 (8)	0.0044 (9)	−0.0020 (9)
C1G1	0.0453 (12)	0.0557 (13)	0.0500 (13)	−0.0058 (11)	0.0180 (11)	−0.0036 (11)
C1D	0.0454 (12)	0.0660 (16)	0.0611 (16)	0.0172 (11)	0.0121 (12)	−0.0050 (12)
C1B2	0.0371 (10)	0.0398 (9)	0.0329 (10)	0.0042 (8)	−0.0012 (8)	0.0026 (8)
C1G2	0.0551 (13)	0.0404 (11)	0.0493 (12)	0.0171 (10)	0.0039 (11)	−0.0012 (9)

### Geometric parameters (Å, º)

**Table:**

C1—N2	1.337 (3)	C1B—H1B6	0.95 (3)
C1—C6	1.391 (3)	C1'—O1	1.180 (3)
C1—H1	0.91 (2)	C1'—O2	1.297 (3)
N2—C3	1.334 (3)	O2—H21	0.93 (5)
C3—C4	1.375 (3)	C1B1—C1G1	1.521 (3)
C3—H2	0.91 (3)	C1B1—H1B2	0.97 (2)
C4—N3	1.331 (3)	C1B1—H1B1	1.03 (2)
C4—H3	1.00 (3)	C1G1—C1D	1.525 (4)
N3—C6	1.329 (3)	C1G1—H1G1	0.98 (3)
C6—C0	1.514 (3)	C1G1—H1G2	0.90 (3)
C0—O0'	1.221 (2)	C1D—C1G2	1.505 (4)
C0—N1	1.336 (3)	C1D—H1D2	1.01 (3)
N1—C1A	1.478 (2)	C1D—H1D1	1.00 (3)
N1—H1N	0.79 (2)	C1B2—C1G2	1.523 (3)
C1A—C1B1	1.532 (3)	C1B2—H1B4	1.00 (2)
C1A—C1B	1.539 (2)	C1B2—H1B3	0.97 (3)
C1A—C1B2	1.540 (3)	C1G2—H1G4	0.97 (3)
C1B—C1'	1.509 (3)	C1G2—H1G3	1.01 (3)
C1B—H1B5	0.91 (3)		
			
N2—C1—C6	121.03 (19)	O1—C1'—C1B	126.5 (2)
N2—C1—H1	117.5 (14)	O2—C1'—C1B	112.51 (18)
C6—C1—H1	121.3 (14)	C1'—O2—H21	106 (3)
C3—N2—C1	116.57 (18)	C1G1—C1B1—C1A	112.85 (17)
N2—C3—C4	122.0 (2)	C1G1—C1B1—H1B2	113.6 (14)
N2—C3—H2	118.2 (16)	C1A—C1B1—H1B2	109.0 (14)
C4—C3—H2	119.8 (16)	C1G1—C1B1—H1B1	108.9 (12)
N3—C4—C3	121.9 (2)	C1A—C1B1—H1B1	104.3 (12)
N3—C4—H3	117.3 (15)	H1B2—C1B1—H1B1	107.7 (18)
C3—C4—H3	120.8 (15)	C1B1—C1G1—C1D	110.9 (2)
C6—N3—C4	116.44 (19)	C1B1—C1G1—H1G1	110.5 (15)
N3—C6—C1	122.0 (2)	C1D—C1G1—H1G1	111.0 (15)
N3—C6—C0	118.85 (18)	C1B1—C1G1—H1G2	112.3 (19)
C1—C6—C0	119.08 (17)	C1D—C1G1—H1G2	111.1 (19)
O0'—C0—N1	126.08 (19)	H1G1—C1G1—H1G2	101 (2)
O0'—C0—C6	119.79 (18)	C1G2—C1D—C1G1	111.1 (2)
N1—C0—C6	114.12 (17)	C1G2—C1D—H1D2	106.1 (16)
C0—N1—C1A	126.55 (16)	C1G1—C1D—H1D2	111.2 (16)
C0—N1—H1N	115.3 (17)	C1G2—C1D—H1D1	107.4 (17)
C1A—N1—H1N	116.8 (17)	C1G1—C1D—H1D1	113.3 (18)
N1—C1A—C1B1	111.06 (16)	H1D2—C1D—H1D1	107 (2)
N1—C1A—C1B	109.15 (15)	C1G2—C1B2—C1A	113.00 (17)
C1B1—C1A—C1B	108.90 (15)	C1G2—C1B2—H1B4	110.6 (13)
N1—C1A—C1B2	106.91 (15)	C1A—C1B2—H1B4	109.3 (13)
C1B1—C1A—C1B2	108.81 (16)	C1G2—C1B2—H1B3	110.4 (15)
C1B—C1A—C1B2	112.02 (16)	C1A—C1B2—H1B3	102.9 (15)
C1'—C1B—C1A	115.79 (16)	H1B4—C1B2—H1B3	110 (2)
C1'—C1B—H1B5	106.5 (16)	C1D—C1G2—C1B2	112.6 (2)
C1A—C1B—H1B5	108.4 (17)	C1D—C1G2—H1G4	109.7 (17)
C1'—C1B—H1B6	108.0 (15)	C1B2—C1G2—H1G4	107.0 (16)
C1A—C1B—H1B6	107.2 (15)	C1D—C1G2—H1G3	111.0 (17)
H1B5—C1B—H1B6	111 (2)	C1B2—C1G2—H1G3	109.4 (16)
O1—C1'—O2	121.0 (2)	H1G4—C1G2—H1G3	107 (2)
			
C6—C1—N2—C3	−1.5 (3)	C0—N1—C1A—C1B2	−173.2 (2)
C1—N2—C3—C4	0.8 (3)	N1—C1A—C1B—C1'	55.0 (2)
N2—C3—C4—N3	0.3 (4)	C1B1—C1A—C1B—C1'	176.42 (19)
C3—C4—N3—C6	−0.6 (3)	C1B2—C1A—C1B—C1'	−63.2 (2)
C4—N3—C6—C1	−0.2 (3)	C1A—C1B—C1'—O1	23.6 (4)
C4—N3—C6—C0	177.82 (19)	C1A—C1B—C1'—O2	−157.9 (2)
N2—C1—C6—N3	1.3 (3)	N1—C1A—C1B1—C1G1	−62.6 (2)
N2—C1—C6—C0	−176.68 (19)	C1B—C1A—C1B1—C1G1	177.20 (19)
N3—C6—C0—O0'	162.9 (2)	C1B2—C1A—C1B1—C1G1	54.8 (2)
C1—C6—C0—O0'	−19.1 (3)	C1A—C1B1—C1G1—C1D	−57.0 (3)
N3—C6—C0—N1	−18.5 (3)	C1B1—C1G1—C1D—C1G2	55.1 (3)
C1—C6—C0—N1	159.53 (19)	N1—C1A—C1B2—C1G2	67.3 (2)
O0'—C0—N1—C1A	8.4 (4)	C1B1—C1A—C1B2—C1G2	−52.7 (2)
C6—C0—N1—C1A	−170.04 (17)	C1B—C1A—C1B2—C1G2	−173.13 (18)
C0—N1—C1A—C1B1	−54.6 (2)	C1G1—C1D—C1G2—C1B2	−53.8 (3)
C0—N1—C1A—C1B	65.5 (2)	C1A—C1B2—C1G2—C1D	53.7 (3)

### Hydrogen-bond geometry (Å, º)

**Table:**

*D*—H···*A*	*D*—H	H···*A*	*D*···*A*	*D*—H···*A*
O2—H21···N2^i^	0.93 (4)	1.86 (4)	2.791 (3)	177 (4)
N1—H1*N*···N3	0.79 (2)	2.34 (2)	2.729 (2)	111.3 (19)

Symmetry code: (i) −*x*+3/2, *y*, *z*+1/2.
